# The Inclusion of Sex and Gender Beyond the Binary in Toxicology

**DOI:** 10.3389/ftox.2022.929219

**Published:** 2022-07-22

**Authors:** Dillon E. King

**Affiliations:** ^1^ Integrated Toxicology and Environmental Health, Nicholas School of the Environment, Duke University, Durham, NC, United States; ^2^ Division of Reproductive Sciences, Department of Obstetrics and Gynecology, Duke University Medical Center, Durham, NC, United States

**Keywords:** sex differences, gender, intersex, toxicology, health equity

## Definitions

Assigned sex is the label given at birth by medical professionals based on an individual’s chromosomes, hormone levels, sex organs, and secondary sex characteristics. As a note, the term “biologic sex” is understood by many to be an outdated term, due to its longstanding history of being used to invalidate the authenticity of trans identities. Although sex is typically misconceptualized as a binary of male (XY) or female (XX), many other chromosomal arrangements, inherent variations in gene expression patterns, and hormone levels exist. Intersex categorizations include variations in chromosomes present, external genitalia, gonads (testes or ovaries), hormone production, hormone responsiveness, and internal reproductive organs. Medical classification of intersex individuals is not always done at birth, as many intersex traits do not become apparent until puberty or later in life. Currently, there are at least 40 known variations that fall into intersex classifications (Carpenter, 2018). Notably, complex biologic variations can occur in everyone, and sex may best be viewed as a spectrum comprised of many traits. Gender is widely understood to be distinguished from sex and is an experienced aspect of self and a social construct of norms, behaviors, and roles that varies between societies and over time.

## Introduction

The inclusion of sex as a biologic variable is critical to understanding how individuals’ health and disease outcomes vary (Gochfeld, 2017; Shannon et al., 2019; Garcia-Sifuentes and Maney, 2021). When reading scientific papers, I often examine whether the study was completed in males or females, if the researchers specified sex, or if they analyzed for sex differences. Often, the sex of the model organism or cell line has not been disclosed, or if it has, only XY-derived samples were used (Beery and Zucker, 2011; Miller, 2012; Shah et al., 2013; Park et al., 2015; Sugimoto et al., 2019; Garcia-Sifuentes and Maney, 2021; Kim et al., 2021). Prioritization of XY subjects in research leaves anyone not a cisgender male—“cisgender” refers to individuals whose gender identity aligns with the sex assigned to them at birth—at much greater uncertainty regarding health outcomes (Weisman and Cassard, 1999; Mazure and Jones, 2015; Duffy et al., 2020; Woitowich et al., 2020).

In 2016, the NIH mandated that sex be included as a biologic variable in scientific studies (National Institutes of Health (NIH), 2015). While this action has proven beneficial and successful (Arnegard et al., 2020), we should continue to encourage individual fields of study like toxicology to rigorously assess for differences in susceptibility to chemicals and health outcomes by intersectional variables (Crenshaw, 1989) such as sex, race, gender, etc., to ensure proper protective measures for everyone. Our efforts to identify members of society vulnerable to environmental exposures should include assessing sex as a biologic variable beyond the binary of male and female, and analysis of gender should include considerations of trans, non-binary, and gender non-conforming individuals.

## On Sex and the Inclusion of Intersex Individuals in Research

Moving forward, we should consider implications of sex beyond the binary categories of male (XY) and female (XX). Even within XX and XY individuals, one’s lifetime endogenous and exogenous hormone milieu is a spectrum. This confluence of genetics and hormonal variations should be considered in experimental design, as hormone influence can be crucial to the field of mechanistic toxicology, as illustrated by the role of estrogens in promoting cancer (Brown and Hankinson, 2015). We need to look beyond evaluating only male and female models by including models that represent intersex individuals. Reporting on intersex frequencies is both scarce and controversial. It is routinely cited that intersex individuals are estimated to comprise 1%–2% of the US population (Fausto-Sterling, 1993; Fausto-Sterling, 2000; Pappas and Migeon, 2017). This percentage includes diagnoses such as Klinefelter syndrome, Turner syndrome, and late-onset adrenal hyperplasia, which many argue should not be included in the definition of intersex for frequency reporting purposes given their lack of association with ambiguous genitalia (Sax, 2002; Griffiths, 2018). When excluding these conditions, intersex individuals are then believed to comprise 0.02% of the US population (Sax, 2002). Even when we assume the conservative estimate of 0.02%, this corresponds to 1 in 5,000 people. For context, that frequency is similar to people known to have genetic mitochondrial diseases (Lightowlers et al., 2015), which has been recognized as needing to be studied in the context of environmental exposures (Meyer et al., 2013). Why has this level of concern not been extended to the intersex population? Even in the field of sex differences, few people study the relationship between the many intersex variations and health. By not studying intersex individuals at the basic biological level or in the context of toxicology, we fail to ensure their safety.

From an epidemiologic standpoint, improvements in data collection and analysis can help us identify if these individuals are more vulnerable to various environmental exposures. Improvements with regard to characterizing and reporting on the frequency of intersex variations as well as the inclusion of intersex individuals in larger studies can help us to identify health disparities. Questionnaires including adequate gender (Adams et al., 2017; Garrett-Walker and Montagno, 2021; Kronk et al., 2021; Miyagi et al., 2021) and sex of the subject population can improve and enhance stratification of populations in data analysis related to health endpoints and environmental exposures, but should be done in collaboration with advocacy groups that strive to represent intersex individuals, such as the Intersex Society of North America, InterACT, or The Intersex Justice Project (Bolte et al., 2021; Richardson, 2022). The inclusion of advocacy groups in study design is crucial to ensuring equity and respect in research design. We should not seek to pathologize intersex individuals in these endeavors but listen to them and provide them with the knowledge of their health that they need and want. I acknowledge that inclusion in epidemiologic studies is not a quick fix, as enriching studies with enough individuals to enable sufficient statistical power will be difficult. Inclusion of trans, non-binary, and intersex individuals in biomedical research poses a large challenge in that asking people to disclose information regarding their sex and gender identity beyond what is typically in medical records may impede recruitment for clinical research, given the social stigma.

Because intersex refers to a variety of biologic differences, this also poses a particular challenge for *in vitro* systems. Without deeper characterization of the intersex population and potential differences in sensitivity to hormones and other agents, there is a potential gap in risk assessment for these populations in terms of how gender-affirming healthcare and environmental agents can influence their health. Part of the goal of toxicology is to characterize mechanisms of action for compounds in potentially sensitive subpopulations and ensure proper safety factors are in place in risk assessment (Pettit, 2020). Given that toxicologists need to be able to assess chemical safety quickly with the large amount of chemicals on the National Toxicity Program (NTP) priority list, it is important that we have model systems that can be generalizable to everyone when screening for chemical safety. While it will require additional time and financial resources to include the proper variables to investigate health and toxicology concerns in a way inclusive of all intersex individuals, it is crucial that we strive to investigate health in service to everyone so all people can receive high-quality medical care.

## On the Inclusion of Gender in Research

Social factors like gender strongly influence the chemicals individuals are exposed to and their medical care following exposure (Gochfeld, 2007; Mauvais-Jarvis et al., 2020; Goldsmith and Bell, 2021). Much like we recognize occupational hazards of certain jobs, such as higher pesticide exposure of agriculture workers, we should recognize that gender norms can greatly influence someone’s exposome. As toxicologists, we need to also think about how we select chemicals to study in equitable ways that ensure people of all genders are accounted for. If cis-women and trans-women are more likely to use personal care products given the societal norms involved in hair, skin, and nail care, we should prioritize studying the compounds found in these products and talking in depth about the hormonally active compounds these products contain. By including people of all genders in exposomic studies, we can begin to investigate how people of different genders may have different levels of exposures to various compounds.

An individual’s gender also alters their interactions with medical professionals, given that laws and social attitudes alter the access to and quality of healthcare services received by intersex, transgender, and nonbinary individuals (Mauvais-Jarvis et al., 2020; Ashley and Domínguez, 2021). Many studies have documented differential medical care for patients experiencing the same symptoms, which points to race and gender bias in the medical field (FitzGerald and Hurst, 2017). What does this differential medical care mean for someone who has been exposed to something toxic and needs immediate medical attention--or the more likely scenario, is seeking answers for chronic symptoms? If transgender and nonbinary individuals feel alienated from the medical field, will they seek medical attention? If they do, will they receive high-quality medical care? The need to investigate the interplay of the influence of both sex and gender differences in the experience and treatment of lung disease has been addressed (Silveyra et al., 2021), with considerable contributions from the COPD field (Raghavan et al., 2017). We should continue to encourage researchers to investigate how gender and society influence the exposome, diagnosis, and medical treatment of individuals.

Additionally, some trans and nonbinary people may take gender-affirming hormone treatments intentionally designed to alter their biological systems and improve mental health outcomes (Green et al., 2021). Transparent communication of health risks associated with hormone treatments is crucial to ensuring the health of all individuals. While there are known health risks associated with hormone therapies, other potential risks remain unstudied (Tassinari and Maranghi, 2021b). The lack of comprehensive knowledge on the risks associated with hormone therapies may be a serious concern with regard to exposure to endocrine disrupting compounds (EDCs) (Tassinari and Maranghi, 2021a), because hormone treatments may alter susceptibility to certain environmental chemicals such as EDCs. In these cases, patients should be advised in order to make informed medical decisions. Importantly, these risks should not be used to dissuade people from receiving gender-affirming healthcare. The medical field is responsible for providing the necessary information for informed consent, prioritizing preventative measures such as increased medical screenings, and education on ways to reduce individual risk. It is also possible that environmental exposures to compounds like EDCs could alter the effectiveness of gender-affirming treatments. It is conceivable that estrogenic compounds such as phenols and parabens commonly found in personal care products could interfere with treatments in which someone is taking estrogen blockers and testosterone. Studies are needed to investigate the role of environmental exposures and their impact(s) on gender-affirming health care. With this in mind, we must ensure that medical research includes males, females, trans individuals, and intersex individuals, and that sex and gender are studied in the context of toxicology and human health from an intersectional lens.

## Moving Forward

The 2019–2023 Trans-NIH Strategic Plan for Women’s Health Research is a powerful statement promoting recognition of the need to address the influence of both sex and gender on human health and promoting equitable health for all (Office of Research on Women’s Health (ORWH), 2019). However, this document fails to recognize the critical need to include trans, nonbinary, and intersex individuals (Greaves and Ritz, 2022). In the field of toxicology, time, energy, and resources are dedicated to understanding how humans interact with their environment and how these interactions influence their health. Human-environment interaction goes beyond genetics. The complex and intersectional issues of race, socioeconomics, gender, etc., that humans face daily influences how they interact with their environments, their consequent exposures, their bodies’ response to those exposures, and the healthcare they receive. Moving forward, we must address the complexities and interactions between our social environment and physical environment in addition to our genetics and hormone exposures and include these considerations in our study designs.
